# Are (poly)phenols contained in 100% fruit juices mediating their effects on cardiometabolic risk factors? A meta-regression analysis

**DOI:** 10.3389/fnut.2023.1175022

**Published:** 2023-06-16

**Authors:** Agnieszka Micek, Walter Currenti, Cristiana Mignogna, Alice Rosi, Ignazio Barbagallo, Ali A. Alshatwi, Daniele Del Rio, Pedro Mena, Justyna Godos

**Affiliations:** ^1^Department of Nursing Management and Epidemiology Nursing, Institute of Nursing and Midwifery, Jagiellonian University Medical College, Krakow, Poland; ^2^Department of Biomedical and Biotechnological Sciences, University of Catania, Catania, Italy; ^3^Human Nutrition Unit, Department of Food and Drug, University of Parma, Parma, Italy; ^4^Department of Food Science and Nutrition, College of Food and Agricultural Sciences, King Saud University, Riyadh, Saudi Arabia

**Keywords:** polyphenols, anthocyanins, fruit juice, blood preasure, blood lipids, blood glucose, metabolic

## Abstract

**Background:**

The consumption of 100% fruit juices has not been associated with substantial detrimental outcomes in population studies and may even contribute to improving the cardiometabolic profile if included in a healthy balanced diet. The main contributors to such potential beneficial effects include vitamins, minerals, and likely the (poly)phenol content. This study aimed to investigate whether the (poly)phenols contained in 100% fruit juices may mediate their effects on cardiometabolic risk factors based on published randomized controlled trials (RCT).

**Methods:**

A systematic search in PubMed/MEDLINE and Embase, updated till the end of October 2022, was carried out to identify RCT providing quantitative data on (poly)phenol content in 100% fruit juices and used as an intervention to improve cardiometabolic parameters such as blood lipids, glucose, and blood pressure. Meta-regression analysis was performed to calculate the effect of the intervention [expressed as standardized mean difference and 95% confidence intervals (CI)] using the (poly)phenol content as moderator.

**Results:**

A total of 39 articles on RCT investigating the effects of 100% fruit juices on cardiometabolic risk factors reporting data on total (poly)phenol and anthocyanin content were included in the analysis. Total (poly)phenol content was substantially unrelated to any outcome investigated. In contrast, each 100 mg per day increase in anthocyanins was related to 1.53 mg/dL decrease in total cholesterol (95% CI, −2.83, −0.22, *p* = 0.022) and 1.94 mg/dL decrease in LDL cholesterol (95% CI, −3.46, −0.42, *p* = 0.012). No other potential mediating effects of anthocyanins on blood triglycerides, glucose, systolic and diastolic pressure were found, while a lowering effect on HDL cholesterol after excluding one outlier study was observed.

**Discussion:**

In conclusion, the present study showed that anthocyanins may mediate the potential beneficial effects of some 100% fruit juices on some blood lipids. Increasing the content of anthocyanins through specific fruit varieties or plant breeding could enhance the health benefits of 100% fruit juices.

## 1. Introduction

Over the last decades, dietary (poly)phenols have been a focus of major interest due to their potential health benefits. This heterogeneous group of molecules is widely spread in the plant kingdom and is commonly found in fruits and vegetables. Depending on their chemical structure, they can be classified as flavonoids and non-flavonoids as the two major groups, but there is a great variety of molecules with many diverse properties and functions within each family (1). Regarding research on humans, there is growing evidence from observational studies showing a lower risk of incidence and mortality from cardiovascular diseases (CVD) associated with higher intakes of total and major classes of flavonoids, such as flavonols, flavones, flavanones, anthocyanins, and flavan-3-ols (2, 3). Epidemiological evidence suggests that higher dietary intakes of flavonoids, particularly from fruits, may have beneficial effects on the risk of CVD incidence and mortality (2, 3), type 2 diabetes incidence (4), and hypertension (5). One of the key dietary sources of flavonoids is fruit, and a recent meta-analysis reports a 10% lower risk of CVD with each 100 g/day increased fruit intake, peaking at 300 g/day for ischemic heart disease risk (6). Moreover, studies on specific fruits, such as citrus and berries, reported stronger positive associations for cardiovascular prevention (7). The observed evidence may provide a rationale for including (poly)phenol-rich foods and beverages in the recommended diet as a potential strategy to prevent CVD.

The consumption of 100% fruit juices is generally considered a secondary choice compared with whole fruits. One reason for this view is the loss of fiber when juice is extracted from the fruit (8). Country-specific dietary guidelines vary considerably regarding the place of 100% fruit juices in a healthy balanced diet, ranging from advice to avoid them to counting one daily serving of juice as a serving of fruit (9). However, since compliance with whole fruit consumption in the general population is relatively low (10), the consumption of 100% fruit juices could still be considered an appealing and cost-effective alternative when whole fruit consumption is not possible. Moreover, previous studies showed that overall fruit and vegetable consumption contributes up to one third of daily fiber intake in low whole-grain consumers, but reaches around one fifth in adequate whole-grain consumers (11–13). This suggests that the major contribution to dietary fiber is not fruit but other dietary sources, such as whole grains. Hence, it is possible that components of fruits other than dietary fiber may play a role in preventing CVD.

Another reason why 100% fruit juice is viewed as an inferior choice to whole fruit is associated with the classification of the natural sugars in fruit juices, but not whole fruits, as free sugars (14). Dietary recommendations suggest limiting free sugars regardless of their source in the diet. While there is undisputed evidence that high consumption of sugar-sweetened beverages, a major source of free sugars, is detrimental to metabolic health and body weight control (15), a meta-analysis of randomized controlled trials (RCT) revealed that higher consumption of 100% fruit juices does not increase the risk of cardiometabolic risk factors. On the contrary, the study reported null effects on body weight, blood lipids, and glucose metabolism, while a beneficial effect toward blood pressure and arterial compliance was found (16). However, no data have been reported on potential components of 100% fruit juices that may exert possible beneficial effects, or at least counterbalance the presence of free sugars. With this hypothesis, we aimed to explore whether the content of (poly)phenols may mediate or modify the effects of 100% fruit juices on major cardiometabolic risk factors in dietary intervention trials.

## 2. Methods

### 2.1. Systematic search and study selection

A systematic search for all studies examining interventions with (poly)phenol-containing 100% fruit juices and their effects on cardiometabolic biomarkers was performed using PubMed/MEDLINE and Embase from their inception until the end of August 2022 and updated till the end of October 2022. The search strategy was based on combining the relevant keywords related to 100% fruit juices and cardiometabolic risk factors used in combination as MeSH terms and text words (Supplementary Table 1). Reference lists of eligible studies were also examined for any additional studies not previously identified. If more than one study reporting results from the same trial was retrieved, only the study including the most comprehensive data was included in the meta-regression analysis. Studies that provided insufficient statistical data were excluded. The systematic search and study selection were performed by two independent authors (A.M. and J.G.). The design, analysis, and reporting of this study followed the Preferred Reporting Items for Systematic Reviews and Meta-Analyses (PRISMA) guidelines. Studies were eligible if they met the following inclusion criteria: (i) randomized controlled trials with the independent-group pre-test–post-test design reporting on the changes in cardiometabolic risk factors (blood pressure, blood lipid profile, and blood glucose levels); (ii) studies evaluating the effect of the intervention with 100% fruit juice; (iii) studies reporting the (poly)phenolic content of the 100% fruit juice; (iv) studies with control drinks not containing (poly)phenols; (v) studies exploring long-term effects of the intervention (at least 1 week); and (vi) studies reporting on adult populations and not reporting on patients with end-stage degenerative diseases, or on pregnant women. The study protocol was registered in the PROSPERO International Prospective Register of Systematic Reviews database (ID number: CRD42022339493).

### 2.2. Data extraction and quality assessment

Data from all included studies were extracted using a standardized electronic form. The following information was collected: first author name, publication year, study design and location, population age and gender, sample size and intervention duration, type of intervention and its main characteristics (including phenolic content), type of comparator, details on the outcome of interest, and measures needed to calculate size effects for each intervention at the beginning and at the end of the trial. The Cochrane risk of bias tool was used to evaluate the quality of included studies (17). Two investigators assessed the methodological quality independently, and any incongruity was resolved by consensus (A.M. and J.G.).

### 2.3. Statistical analyses

Analyses were conducted separately on studies with different cardiometabolic risk factor measurements and for different (poly)phenol types. Within each measurement, all results were converted into standard units, and mean differences (MD) of pre-post changes between the two intervention groups (juice vs. control) were calculated. In reports which fail to provide sufficient data for computing effect size estimates properly accounting for the paired nature of the design, the correlation between measurements before and after each intervention was imputed at level 0.5, whereas the correlation of change-from-baseline measures between active and placebo treatment periods was set at 0 (18). Finally, effect sizes were harmonized using a random-effects model with DerSimonian and Laird estimator of between-study variance. Heterogeneity was assessed by the *I*^2^ statistic and formally complemented by the Cochran Q-test under a level of significance set at 0.1. Publication bias was verified by visual inspection of funnel plots for asymmetry and by quantitative method, Egger’s regression test. Pooled results were reported as MD with 95% confidence intervals (CI) with two-sided *p* values, with values of *p* less than 0.05 considered statistically significant. To then verify whether the retrieved effects were associated with the (poly)phenol content in 100% fruit juices, the daily amount of (poly)phenol [meaning (poly)phenol content in the intervention arm] was treated as a moderator of the association between intervention and cardiometabolic risk factors measurements. Therefore, meta-regression analyses were conducted with the total and specific (poly)phenol content in the intervention arm additionally incorporated into the models. The significance and sign of the slope coefficient of the meta-regression line were tested to show the direction and strength of the dose–response relationship. A sensitivity analysis was conducted to assess the stability of results by testing alternative models excluding one study each time and pooling estimates for the rest of the studies. Subgroup analyses by health status of participants were performed. All analyses were performed with R software (Development Core Team, Vienna, Austria, version 4.0.4).

## 3. Results

### 3.1. Main characteristics of the included studies

**Figure 1:**
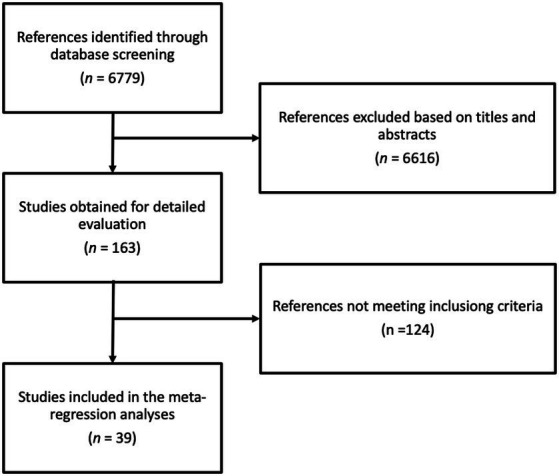
Flow chart of study selection process.

Figure 1 shows the process of the literature search and selection. The initial database search identified 6,779 potential articles, of which 6,616 were excluded based on title and abstract assessment leaving 163 articles. After full-text examination, 124 articles were identified as ineligible based on one or more of the following reasons: (i) inadequate intervention, (ii) inadequate or lack of comparator, (iii) reporting on acute effects, (iv) providing insufficient statistics, (v) not providing phenolic content of the intervention, and (vi) inadequate study design. Finally, 39 articles met the inclusion criteria (19–57).

The main characteristics of the 25 parallel (19–24, 26, 29, 30, 32, 35, 36, 38, 39, 41, 43–45, 48, 50, 51, 54–57) and 14 crossover (25, 27, 28, 31, 33, 34, 37, 40, 42, 46, 47, 49, 52, 53) RCT on 100% fruit juices and cardiometabolic outcomes are provided in Table 1. Eligible studies reported on the following juices: pomegranate, cranberry, tart cherry, Concord grape, blueberry, blood orange, chokeberry, bayberry, strawberry, blackcurrant, *Aronia melanocarpa*, plum, and mixed berry juices. Studies conducted on other 100% fruit juices (i.e., orange juices) did not provide enough data to actually perform analyses on specific (poly)phenol contained (i.e., flavanones and flavan-3-ols) while available data was retrieved for anthocyanins. Included studies involved adult participants, being at low and high cardiovascular risk. The intervention duration varied from 1 to 16 weeks. Most of the trials provided measures on more than one of the investigated outcomes, including blood pressure (*n* = 32), blood lipids (*n* = 32), and blood glucose levels (*n* = 27). The risk of bias assessment showed that when considering overall risk of bias, the majority of the studies were subjected unclear risk of bias (Supplementary Figures 1, 2).

**Table 1:** Main characteristics of the randomized clinical trials on 100% fruit juices and cardiometabolic outcomes.

Author, year, country	Participant characteristics	Sex; mean age	Design	Duration of intervention	Intervention	Comparison	Intervention juice phenolic content (concentration)	Outcomes of interest
Cerdá (19), Spain	30 patients with chronic obstructive pulmonary disease	M; I: 60 years, C: 63.4 years	Parallel	5 weeks	Pomegranate juice (400 mL/day)	Placebo beverage (400 mL/day)	Polyphenols: 6,650 mg/L	LP, GP
							Anthocyanins: 475 mg/L	
Duthie (20), Scotland	20 healthy volunteers	F; I: 27.3 years, C: 28.3 years	Parallel	2 weeks	Cranberry juice (750 mL/day)	Placebo beverage (750 mL/day)	Polyphenols: 1,136 mg/L	LP
							Anthocyanins: 2.80 mg/L	
Hollis (21), United States	51 overweight subjects	MF; I: 22 years, C: 26 years	Parallel	12 weeks	Concord grape juice (480 mL/day)	Placebo beverage (480 mL/day)	Polyphenols: 1,945 mg/L	LP, GP
							Anthocyanins: 398 mg/L	
Park (22), Korea	40 subjects with borderline hypertension	M; I: 43 years, C: 46 years	Parallel	8 weeks	Concord grape juice (5.5 mL/kg/day)	Placebo beverage (5.5 mL/kg/day)	Polyphenols: 2,108 mg/L	LP, BP
Basu (23), United States	48 obese individuals with metabolic syndrome	MF; I: 51.5 years, C: 48 years	Parallel	8 weeks	Blueberry juice (960 mL/day)	Water (960 mL/day)	Polyphenols: 1,692 mg/L	LP, GP, and BP
							Anthocyanins: 773 mg/L	
Basu (24), United States	27 subjects with metabolic syndrome	MF; I: 48 years, C: 45 years	Parallel	8 weeks	Strawberry juice (960 mL/day)	Water (960 mL/day)	Polyphenols: 2,089 mg/L	LP, GP, and BP
							Anthocyanins: 160 mg/L	
Dohadwala (25), United States	64 patients with prehypertension and stage 1 hypertension	MF; 43 years	Crossover	8 weeks	Concord grape juice (7 mL/kg/day)	Placebo beverage (7 mL/kg/day)	Polyphenols: 1,970 mg/L	LP, GP, and BP
Basu (26), United States	31 subjects with metabolic syndrome	F; 52 years	Parallel	8 weeks	Cranberry juice (480 mL/day)	Placebo beverage (480 mL/day)	Polyphenols: 954 mg/L	LP, GP, and BP
							Anthocyanins: 52 mg/L	
Dohadwala (27), United States	44 patients with coronary artery heart disease	MF; 62 years	Crossover	4 weeks	Cranberry juice (480 mL/day)	Placebo beverage (480 mL/day)	Polyphenols: 1,740 mg/L	LP, GP, and BP
							Anthocyanins: 196 mg/L	
Buscemi (28), Italy	19 subjects with increased cardiovascular risk	MF; I: 48 years, C: 35 years	Crossover	7 days	Red orange juice (500 mL/day)	Placebo beverage (500 mL/day)	Polyphenols: 419 mg/L	GP
							Anthocyanins: 71.3 mg/L	
Krikorian (29), United States	21 older subjects with mild, age-related memory decline	MF; I: 78 years, C: 75 years	Parallel	16 weeks	Concord grape juice (6.3–7.8 mL/kg/day)	Placebo beverage (6.3–7.8 mL/kg/day)	Polyphenols: 2,091 mg/L	GP, BP
							Anthocyanins: 425 mg/L	
Lynn (30), United Kingdom	48 healthy participants	MF; I: 39 years, C: 36.1 years	Parallel	4 weeks	Pomegranate juice (330 mL/day)	Placebo beverage (330 mL/day)	Polyphenols: 18.6 mmol/L	BP
Tsang (31), United Kingdom	28 overweight or obese volunteers	MF; 50.4 years	Crossover	4 weeks	Pomegranate juice (500 mL/day)	Placebo beverage (500 mL/day)	Polyphenols: 1,685 mg/L	LP, GP, and BP
Flammer (32), United States	69 subjects with peripheral endothelial dysfunction and cardiovascular risk factors	MF; I: 44.8 years, C: 51.4 years	Parallel	4 m	Cranberry juice (460 mL/day)	Placebo beverage (460 mL/day)	Polyphenols: 1,740 mg/L	LP, BP
							Anthocyanins: 151 mg/L	
Ruel (33), Canada	35 healthy overweight participants	M; 45 years	Crossover	4 weeks	Cranberry juice (500 mL/day)	Placebo beverage (500 mL/day)	Polyphenols: 800 mg/L	BP
							Anthocyanins: 42 mg/L	
Guo (34), China	44 participants with features non-alcoholic fatty liver disease	MF; 21.2 years	Crossover	4 weeks	Bayberry juice (500 mL/day)	Placebo beverage (500 mL/day)	Polyphenols: 2,702 mg/L	LP, GP
							Anthocyanins: 835 mg/L	
Khan (35), United Kingdom	64 healthy subjects	MF; I (low): 55 years, I (high): 51 years, C: 51 years	Parallel	6 weeks	Low-polyphenol blackcurrant juice (1,000 mL/day)	Placebo beverage (1,000 mL/day)	Low-polyphenol blackcurrant juice	LP, BP
							Polyphenols: 273 mg/L	
							Anthocyanins: 40 mg/L	
					High-polyphenol blackcurrant juice (1,000 mL/day)		High-polyphenol blackcurrant juice	
							Polyphenols: 815 mg/L	
							Anthocyanins: 143 mg/L	
Lynn (36), United Kingdom	46 healthy subjects	MF; I: 38.3 years, C: 37.2 years	Parallel	6 weeks	Tart cherry juice (250 mL/day)	Placebo beverage (250 mL/day)	Anthocyanins: 1,094 mg/L	LP, BP
Siasos (37), Greece	26 healthy smokers	MF; 26.3 years	Crossover	2 weeks	Concord grape juice (7 mL/kg/day)	Placebo beverage (7 mL/kg/day)	Polyphenols: 1,970 mg/L	LP, GP, and BP
							Anthocyanins: 296 mcmol/L	
Sohrab (38), Iran	44 patients with type 2 diabetes	MF; I: 55 years, C: 56.9 years	Parallel	12 weeks	Pomegranate juice (250 mL/day)	Placebo beverage (250 mL/day)	Polyphenols: 1,946 mg/L	GP
Novotny (39), United States	56 healthy volunteers	MF; I: 49.8 years, C: 51.3 years	Parallel	8 weeks	Cranberry juice (480 mL/day)	Placebo beverage (480 mL/day)	Polyphenols: 720.8 mg/L	LP, GP, and BP
							Anthocyanins: 42.9 mg/L	
Loo (40), Finland	37 patients with mildly elevated blood pressure	MF; 55.8 years	Crossover	8 weeks	Chokeberry juice (300 mL/day)	Placebo beverage (300 mL/day)	Polyphenols: 7,313 mg/L	LP, GP, and BP
							Anthocyanins: 3,413 mg/L	
Kojadinovic (41), Serbia	23 subjects with metabolic syndrome	F; 40–60 years	Parallel	6 weeks	Pomegranate juice (300 mL/day)	Water (not reported)	Polyphenols: 2,938 mg/L	LP, GP, and BP
							Anthocyanins: 21 mg/L	
Moazzen (42), Iran	30 volunteers with metabolic syndrome	MF; 51.6 years	Crossover	1 weeks	Pomegranate juice (500 mL/day)	Placebo beverage (500 mL/day)	Anthocyanins: 100.46 mg/L	LP, GP, and BP
Paquette (43), Canada	41 overweight or obese subjects with insulin resistance	MF; I: 57 years, C: 60 years	Parallel	6 weeks	Strawberry and cranberry juice (120 mL/day)	Placebo beverage (120 mL/day)	Polyphenols: 2,775 mg/L	LP, BP
Chai (44), United States	34 older subjects	MF; I: 70.0 years, C: 69.5 years	Parallel	12 weeks	Tart cherry juice (480 mL/day)	Placebo beverage (480 mL/day)	Polyphenols: 938.8 mg/L	LP, GP, and BP
Bakuradze (45), Germany	57 healthy volunteers	M; I: 23 years, C: 24 years	Parallel	8 weeks	Fruit (red grape, lingonberry, apple, blueberry, strawberry, aronia, and acerola) juice (750 mL/day)	Placebo beverage (750 mL/day)	Polyphenols: 3,600 mg/L	LP
							Anthocyanins: 274.5 mg/L	
Hollands (46), United Kingdom	41 overweight participants	MF; 52.2 years	Crossover	28 days	Blood orange juice (500 mL/day)	Blonde orange juice (500 mL/day)	Anthocyanins: 100 mg/L	LP, GP, and BP
Martin (47), United States	26 overweight or obese participants	MF; 41 years	Crossover	4 weeks	Tart cherry juice (240 mL/day)	Placebo beverage (240 mL/day)	Polyphenols: 4,140 mg/L	LP, GP, and BP
							Anthocyanins: 65 mg/L	
Pokimica (48), Serbia	84 individuals at cardiovascular risk	MF; I (low): 40.8 years, I (high): 42.3 years, C: 39 years	Parallel	4 weeks	Low-polyphenol chokeberry juice (100 mL/day)	Placebo beverage (100 mL/day)	Low-polyphenol chokeberry juice	LP, GP, and BP
							Polyphenols: 2942.8 mg/L	
					High-polyphenol chokeberry juice (100 mL/day)		Anthocyanins: 283 mg/L	
							High-polyphenol chokeberry juice	
							Polyphenols: 11771.1 mg/L	
							Anthocyanins: 1,133 mg/L	
Desai (49), United Kingdom	12 participants with metabolic syndrome	MF; 50 y	Crossover	7 days	Montmorency tart cherry juice (130 mL/day)	Placebo beverage (130 mL/day)	Anthocyanins: 2076.9 mg/L	LP, GP, and BP
do Rosario (50), Australia	31 older adults with cognitive impairment	MF; I (low): 76.1 years, I (high): 75.1 years, C: 74.9 years	Parallel	8 weeks	Low-anthocyanin Queen Garnet Plum juice (250 mL/day)	Apricot juice (250 mL/day)	Low-anthocyanin Queen	BP
							Garnet Plum juice	
							Anthocyanins:188 mg/L	
							High-anthocyanin Queen	
					High-anthocyanin Queen Garnet Plum juice (250 mL/day)		Garnet Plum juice	
							Anthocyanins: 804 mg/L	
Johnson (51), United States	19 individuals with metabolic syndrome	MF; I: 29.3 years, C: 44.2 years	Parallel	12 weeks	Tart cherry juice (480 mL/day)	Placebo beverage (480 mL/day)	Polyphenols: 4458.3 mg/L	LP, GP, and BP
							Anthocyanins: 366.7 mg/L	
Li (52), United Kingdom	15 healthy overweight or obese participants	MF; 28.7 years	Crossover	2 weeks	Blood orange juice (400 mL/day)	Placebo beverage (400 mL/day)	Anthocyanins: 24 mg/L	LP, BP
Richter (53), United States	40 adults with elevated blood pressure	MF; 47 years	Crossover	8 weeks	Cranberry juice (500 mL/day)	Placebo beverage (500 mL/day)	Polyphenols: 674.7 mg/L	LP, GP, and BP
							Anthocyanins: 9.6 mg/L	
Stojković (54), Serbia	54 dyslipidemic individuals	MF; I: 41.1 years, C: 38.5 years	Parallel	4 weeks	*Aronia melanocarpa* juice (100 mL/day)	Placebo beverage (100 mL/day)	Polyphenols: 11771.1 mg/L	LP, GP, and BP
Heiss (55), Germany	44 healthy adults	M; I: 25 years, C: 25 years	Parallel	1 m	Cranberry juice (500 mL/day)	Placebo beverage (500 mL/day)	Polyphenols: 1,050 mg/L	LP, GP, and BP
							Anthocyanins: 108 mg/L	
Hillman (56), United States	20 healthy adults	MF; I: 28 years, C: 27 years	Parallel	30 days	Montmorency tart cherry juice (480 mL/day)	Placebo beverage (480 mL/day)	Polyphenols: 3,304.2 mg/L	BP
							Anthocyanins: 945.8 mg/L	
Sinclair (57), United Kingdom	44 healthy adults	MF; I (cherry): 32.8 years, I (blueberry): 34.1 years, C: 35.1 years	Parallel	20 days	Montmorency tart cherry juice (260 mL/day)	Placebo beverage (260 mL/day)	Montmorency tart cherry juice	LP, GP, and BP
							Anthocyanins: 2,462 mg/L	
							Blueberry juice	
					Blueberry juice (260 mL/day)		Anthocyanins: 2,977 mg/L	

### 3.2. Effect of total (poly)phenol content in 100% fruit juices on cardiometabolic biomarkers

**Figure 2:**
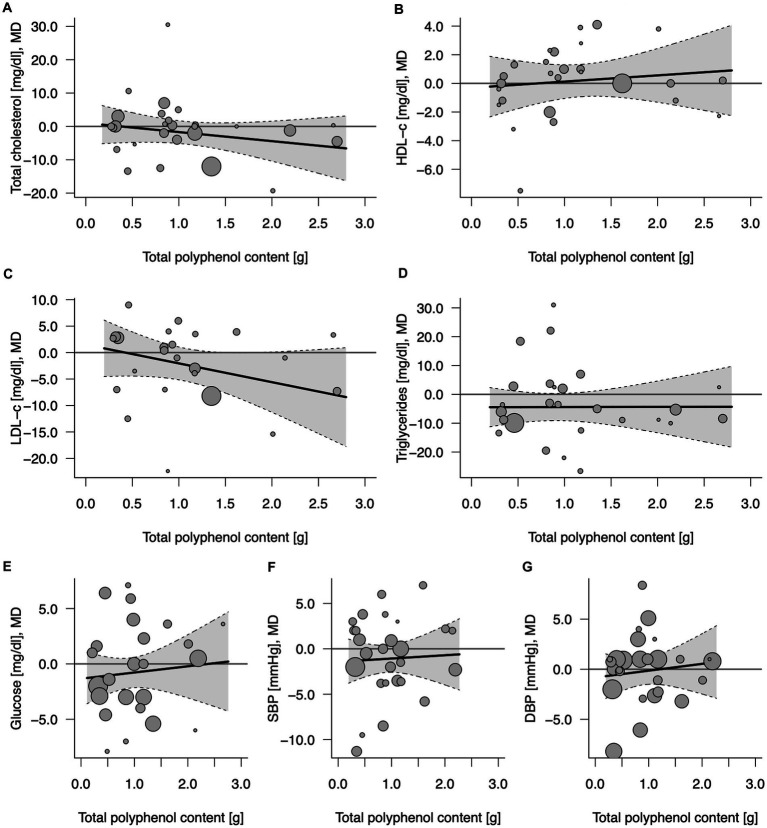
Potential mediating effect of total (poly)phenol content in 100% fruit juice in randomized controlled trials on cardiovascular risk factors: **(A)** Total cholesterol (mg/dL), **(B)** HDL-C (mg/dL), high-density lipoprotein-cholesterol, **(C)** LDL-C (mg/dL), low-density lipoprotein-cholesterol; **(D)** TG (mg/dL), **(E)** Glucose (mg/dL), **(F)** DBP (mmHg), diastolic blood pressure; and **(G)** SBP (mmHg), systolic blood pressure. Solid lines depict regression slopes and reflect how the mean differences in measurement of each specific cardiometabolic biomarker between juice and control change across the (poly)phenol content. Gray shadows represent confidence interval regions for regression slopes. Bubbles reflect observed study-specific mean differences in biomarkers between juice and control and the point sizes are a function of the model weights.

A total of 24–27 comparisons (7–9 from crossover design and 15–20 from parallel design studies, depending on the outcome) were included in the analysis (Table 2). No significant effects of 100% fruit juice intervention on cardiometabolic biomarkers were detected after the meta-analysis of included studies, nor any potential mediating effect of total (poly)phenol content in the intervention groups (Figure 2; Supplementary Figure 3). In some subgroup analyses, a borderline protective activity of juices was observed toward lowering the concentration of LDL cholesterol (LDL-C) in high CVD risk individuals (MD = −3.93, 95% CI: −8.30, 0.43, *p* = 0.077) and significant effect on triglycerides (TG) in individuals with high CVD risk (MD = −9.52, 95% CI: −16.28, −2.76, *p* = 0.006), in trials lasting at least 6 weeks (MD = −6.80, 95% CI: −12.31, −1.28, *p* = 0.016): however, these effects were not related to the total (poly)phenol content (Supplementary Table 2).

**Table 2:** Effect of 100% fruit juice vs. control in randomized controlled trials on cardiovascular risk factors and potential mediating effect of total (poly)phenol content.

		Overall effect of juice vs. control drink				Total polyphenol content effect (per 1 g/day)			
Cardiometabolic biomarker	*n* comparisons	MD (95% CI)	*p*	*I*^2^ [%]	*p*_heter_	ΔMD^&^ (95% CI)	*p*	*I*^2^ [%]	*p*_heter_
*Total cholesterol*	27	−1.85 (−5.25; 1.55)	0.286	0	0.853	−2.72 (−7.94; 2.51)	0.308	0	0.861
*HDL-C*	27	0.20 (−0.93; 1.33)	0.727	0	0.996	0.43 (−1.39; 2.26)	0.640	0	0.995
*LDL-C*	24	−2.24 (−5.33; 0.85)	0.156	0	0.921	−3.55 (−8.61; 1.51)	0.169	0	0.951
*Triglycerides*	26	−4.40 (−9.09; 0.28)	0.066	0	0.944	0.05 (−6.92; 7.02)	0.989	0	0.923
*Glucose*	24	−0.86 (−2.18; 0.46)	0.202	15.6	0.245	0.56 (−1.77; 2.89)	0.636	17.9	0.219
*SBP*	27	−1.11 (−2.57; 0.34)	0.133	16.5	0.223	0.36 (−2.31; 3.03)	0.791	19.4	0.188
*DBP*	27	−0.21 (−1.53; 1.11)	0.757	45.4	0.006	0.68 (−1.80; 3.16)	0.590	45.9	0.006

DBP, diastolic blood pressure; HDL-C, high-density lipoprotein-cholesterol; LDL-C, low-density lipoprotein-cholesterol; MD, mean difference; SBP, systolic blood pressure.

Overall, none of the cardiometabolic biomarkers showed an asymmetrical pattern in the funnel plot that might be indicative of publication bias (Supplementary Figure 4).

### 3.3. Effect of anthocyanin content in 100% fruit juices on cardiometabolic biomarkers

**Figure 3:**
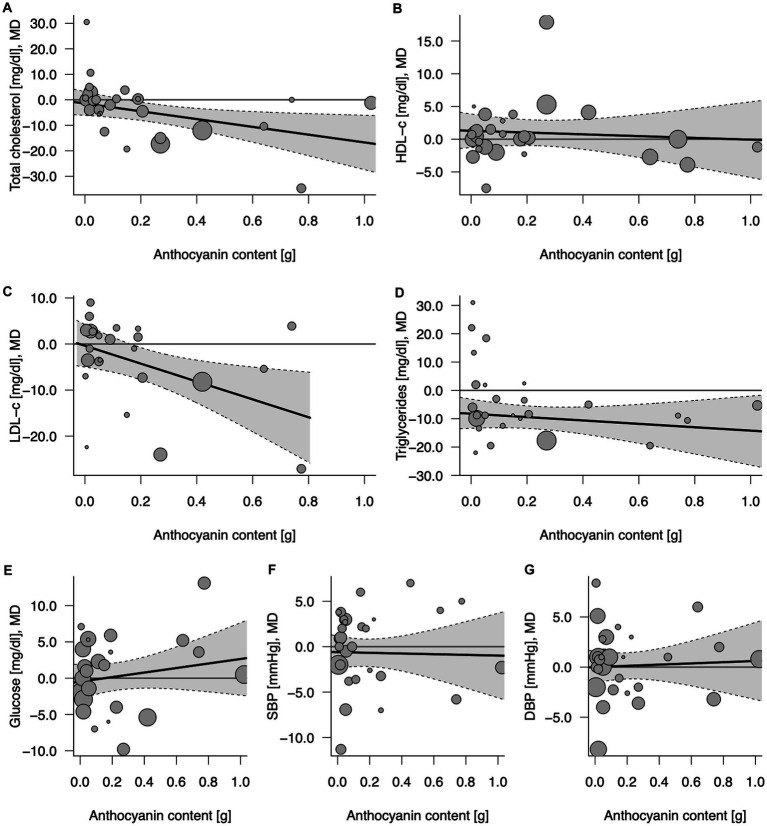
Potential mediating effect of total anthocyanin content in 100% fruit juice in randomized controlled trials on cardiovascular risk factors: **(A)** Total cholesterol (mg/dL), **(B)** HDL-C (mg/dL), high-density lipoprotein-cholesterol, **(C)** LDL-C (mg/dL), low-density lipoprotein-cholesterol; **(D)** TG (mg/dL), **(E)** Glucose (mg/dL), **(F)** DBP (mmHg), diastolic blood pressure; and **(G)** SBP (mmHg), systolic blood pressure. Solid lines depict regression slopes and reflect how the mean differences in measurement of each specific cardiometabolic biomarker between juice and control change across the anthocyanin content. Gray shadows represent confidence interval regions for regression slopes. Bubbles reflect observed study-specific mean differences in biomarkers between juice and control and the point sizes are a function of the model weights.

Eighteen comparisons from randomized clinical trials with repeated measures and parallel design (19–21, 23, 24, 26, 32, 35, 36, 39, 41, 45, 48, 55, 57) and 10 from crossover design (27, 34, 37, 40, 42, 46, 47, 49, 52, 53) depicted the effect of anthocyanins contained in 100% fruit juices on total cholesterol concentration. An overall influence of juice intervention on lipid measurement, regardless of dose anthocyanins consumed with juice, was significant (MD = −4.62, 95% CI: −8.51, −0.72, *p* = 0.020; Table 3; Supplementary Figure 5). The relationship was dose-dependent with the stronger effect for juices containing larger amounts of anthocyanins: each 100 mg/day increase in anthocyanin content was accompanied with 1.53 mg/dL decrease in total cholesterol (95% CI: −2.83, −0.22, *p* = 0.022 per Δanth = +0.1 g/day; Table 3; Figure 3). Introducing the moderator to analysis reduced heterogeneity from 22.6 to 10.4%. Subgroup analysis revealed a beneficial effect of consumption of anthocyanin-rich juice compared to control drink in crossover design studies (MD = −6.67, 95% CI: −11.43, −1.92, *p* = 0.006) with no further effect of anthocyanin content (Supplementary Table 3). On the contrary, in parallel studies, anthocyanin content mediated the juice activity, enhancing the decline of lipid marker levels (ΔMD = −3.35; 95% CI: −5.41, −1.29, *p* = 0.001 per Δanth = +0.1 g/day). Both the overall effects of juice consumption and the effects of anthocyanin content were significant for trials lasting less than 6 weeks (Supplementary Table 3). Moreover, the exclusion of one trial (40), which was identified as an influential point in meta-regression, resulted in a significant slope (ΔMD = −3.55; 95% CI: −6.50, −0.61, *p* = 0.018) in the analysis of crossover studies, confirming additional benefit from the consumption of juices richer in anthocyanins (Supplementary Table 4).

**Table 3:** Effect of 100% fruit juice vs. control in randomized controlled trials on cardiovascular risk factors and potential mediating effect of anthocyanin content.

		Overall effect of 100% fruit juice vs. control				Anthocyanin content effect (per 0.1 g/day)			
Cardiometabolic biomarker	*n* comparisons	MD (95% CI)	*p*	*I*^2^ [%]	*p*_heter_	ΔMD (95% CI)	*p*	*I*^2^ [%]	*p*_heter_
*Total cholesterol*	28	−4.62 (−8.51; −0.72)	0.020	22.6	0.141	−1.53 (−2.83; −0.22)	0.022	10.4	0.311
*HDL-C*	28	0.97 (−0.96; 2.90)	0.324	76.9	<0.001	−0.14 (−0.85; 0.57)	0.708	76.8	<0.001
*LDL-C*	24	−3.97 (−7.97; 0.03)	0.052	34.2	0.052	−1.94 (−3.46; −0.42)	0.012	16.5	0.237
*Triglycerides*	26	−9.55 (−13.52; −5.57)	<0.001	0	0.669	−0.60 (−2.07; 0.88)	0.429	0	0.651
*Glucose*	24	0.22 (−1.63; 2.08)	0.814	45.1	0.009	0.31 (−0.29; 0.91)	0.308	45.5	0.010
*SBP*	29	−0.70 (−2.16; 0.77)	0.353	14.9	0.239	−0.04 (−0.57; 0.49)	0.891	17.9	0.201
*DBP*	29	0.11 (−1.17; 1.39)	0.865	41.4	0.011	0.06 (−0.39; 0.51)	0.792	43.2	0.009

DBP, diastolic blood pressure; HDL-C, high-density lipoprotein-cholesterol; LDL-C, low-density lipoprotein-cholesterol; MD, mean difference; SBP, systolic blood pressure.

There was no evidence of an effect of 100% fruit juices rich in anthocyanins on HDL cholesterol (HDL-C) concentration (Table 3; Figure 3) based on 28 comparisons from RCT [nine from crossover (27, 34, 40, 42, 46, 47, 49, 52, 53) and 19 from parallel design studies (19–21, 23, 24, 26, 32, 36, 39, 41, 45, 48, 51, 55, 57)] for total (MD = 0.97, 95% CI: −0.96, 2.90, *p* = 0.324; *I*^2^ = 76.9%; Supplementary Figure 5) and subgroup analyses (Supplementary Table 3). In the subgroup analysis by study design, the exclusion of one influential study (40) reduced unexplained heterogeneity between crossover trials to a low level (I^2^ = 26.9%) and showed the significant impact of anthocyanins content toward raising the concentration of HDL cholesterol (Supplementary Table 4): the larger amount of anthocyanins in 100% fruit juice was associated with an additional increase in MD between the intervention and the placebo of 1.59 (MD = 1.81, 95% CI: −1.02, 4.64, *p* = 0.210 overall effect and ΔMD = 1.59, 95% CI: 0.34, 2.84, *p* = 0.013 per Δanth = +0.1 g/day).

Twenty-four comparisons [nine from crossover (27, 34, 37, 42, 46, 47, 49, 52, 53) and 15 from parallel design studies (19–21, 23, 24, 26, 39, 41, 45, 48, 51, 55, 57)] were included in the analysis verifying the influence of 100% fruit juice interventions on LDL-C levels. A marginally significant protective activity of 100% fruit juice consumption was observed (MD = −3.97, 95% CI: −7.97, 0.03, *p* = 0.052) with moderate heterogeneity between trials (I^2^ = 34.2%; Table 3; Supplementary Figure 5). The higher amounts of anthocyanins enhanced the LDL cholesterol-lowering effect, which was manifested by a further decline of MD between 100% fruit juice and comparator of −1.94 mg/dL (95% CI: −3.46, −0.42, *p* = 0.012) of LDL cholesterol with each 0.1 g/day increase in the dose of anthocyanins (Figure 3). Simultaneously, a reduction of heterogeneity to 16.5% after introducing the moderator to a model was noted. Subgroups analysis showed a significant dose-dependent impact of juices rich in anthocyanins on LDL cholesterol in studies examining subjects with low CVD risk (ΔMD = −2.72, 95% CI: −4.59, −0.85, *p* = 0.004 per Δanth = +0.1 g/day), trials with follow up shorter than 6 weeks (ΔMD = −2.63, 95% CI: −4.44, −0.83, *p* = 0.004 per Δanth = +0.1 g/day), and the marginally significant result was detected in both crossover trials (ΔMD = −3.44, 95% CI: −6.99, 0.11, *p* = 0.058 per Δanth = +0.1 g/day) and parallel trials (ΔMD = −1.69, 95% CI: −3.39, 0.01, *p* = 0.051 per Δanth = +0.1 g/day; Supplementary Table 3).

Twenty-six comparisons (10 from crossover (27, 34, 37, 40, 42, 46, 47, 49, 52, 53) and 16 from parallel design studies (19–21, 23, 24, 26, 32, 39, 41, 45, 48, 51, 55, 57)) were included in the analysis verifying the influence of 100% fruit juice interventions on TG concentration. A significant impact of juice consumption on TG measurement favoring intervention against the control drink was found, as evidenced by a 9.55 mg/dL larger decrease (MD = −9.55, 95% CI: −13.52, −5.57, *p* < 0.001) in TG during follow-up (Table 3; Supplementary Figure 5), however, with no further effect of anthocyanin content in the beverages (ΔMD = −0.60, 95% CI: −2.07, 0.88, *p* = 0.429 per Δanth = +0.1 g/day; Figure 3). Subgroup analysis showed a significant dose-dependent impact of juices rich in anthocyanins on TG in trials with a follow up <6 weeks (ΔMD = −3.68, 95% CI: −6.82, −0.54, *p* = 0.022 per Δanth = +0.1 g/day). The overall effect of juice was protective toward lowering TG for studies lasting longer than 6 weeks, in both crossover and parallel studies, however, with no further impact of anthocyanin content (Supplementary Table 3). Moreover, after the exclusion of one study (40), meta-regression resulted in a marginally significant slope (ΔMD = −2.24; 95% CI: −4.56, 0.07, *p* = 0.058 per Δanth = +0.1 g/day) showing a tendency toward a stronger impact of juices higher in anthocyanins (Supplementary Table 4).

Twenty-four comparisons [10 from crossover (27, 28, 34, 37, 40, 42, 46, 47, 49, 53) and 14 from parallel design studies (19, 21, 23, 24, 26, 29, 39, 41, 48, 51, 55, 57)] tested the effect of juice interventions on glucose concentration. No evidence of the impact of 100% fruit juices rich in anthocyanins on glucose measurement was detected in the total sample of studies (Table 3; Supplementary Figure 5) nor in subgroup analyses (Supplementary Table 3). In the sensitivity analysis, after the exclusion of the influential study of (40), higher anthocyanin content was associated with a decrease in blood glucose in crossover design trials (ΔMD = −1.89, 95% CI: −3.60, −0.18, *p* = 0.030 per Δanth = +0.1 g/day; Supplementary Table 4).

Finally, there was no evidence of the effect of 100% fruit juices rich in anthocyanins on blood pressure based on 29 comparisons from RCT [10 crossover (27, 33, 37, 40, 42, 46, 47, 49, 52, 53) and 19 parallel design (23, 24, 26, 29, 32, 35, 36, 39, 41, 48, 50, 51, 55–57); Table 3; Supplementary Figure 5] nor in subgroup analyses (Supplementary Table 3).

In the case of cholesterol, HDL and triglycerides signs of an asymmetrical pattern in the funnel plot that might be indicative of publication bias was detected (Supplementary Figure 6).

## 4. Discussion

In this study, we attempted to investigate the role of (poly)phenol content in relation to 100% fruit juice consumption and cardiometabolic risk factors through a meta-regression analysis of RCT. The results showed no significant role of total (poly)phenols in any outcomes investigated. However, a higher content of anthocyanins in 100% fruit juices significantly increased the lowering of total cholesterol and LDL cholesterol; the mediating effects seemed to be stronger in studies that included individuals at high CVD risk (i.e., with metabolic syndrome or multiple cardiovascular risk factors), with a potential additional significant effect also on HDL cholesterol when excluding an outlier study. No further effects were detected on TG, blood glucose or blood pressure. This study adds another dimension to the scientific literature and suggests that (poly)phenols should be taken into account in future dietary intervention trials of 100% fruit juice consumption.

Numerous observational and intervention studies have been conducted to identify the potential impact of 100% fruit juice consumption on such biomarkers, often reporting contrasting results (16). No substantial harm concerning blood glucose and obesity risk has been observed, while a potential protective effect (or an inverse association) was found for blood pressure and the risk of CVD (8). Compared to previous meta-analyses (16) an effect of 100% fruit juice consumption and blood pressure could not be found, probably due to the smaller number of studies included with available data on (poly)phenol content. It has been suggested that the beneficial effects on such cardiometabolic outcomes are related to the potassium content of 100% fruit juices, as this mineral may affect blood pressure and lower the risk of stroke (58, 59). However, none of the research conducted up to date explored the potential mediating effect of other bioactive components in 100% fruit juices, such as (poly)phenols.

Although, in this study, we were not able to demonstrate the role of (poly)phenols in the association between 100% fruit juices and blood pressure or any other outcome, we found that anthocyanins may be potential mediators of improvements in blood lipids in RCT administering 100% fruit juices. Other meta-analyses showed that purified anthocyanin and anthocyanin-rich berry supplementation could significantly reduce blood LDL cholesterol and increase HDL cholesterol (60–63). Moreover, a recent umbrella review concluded that anthocyanins improved plasmatic lipids, glucose metabolism, and endothelial function, without affecting blood pressure in RCT (64). Hence, current evidence is consistent with our findings, suggesting a substantial role of anthocyanins in the observed effects related to 100% fruit juice consumption.

The rationale behind the potential positive effects of (poly)phenols and, specifically, anthocyanins in 100% fruit juices, is supported by the extensive share of scientific literature providing a variety of potential mechanisms. Several preclinical studies conducted *in vitro* or on animals show that (poly)phenols (such as anthocyanins cyanidin-3-glucoside and peonidin-3-glucoside and their metabolites) may affect cellular antioxidant status and inflammation by increasing endogenous antioxidant defenses through activation of genes encoding antioxidant enzymes and modulating various inflammatory pathways (i.e., nuclear factor, erythroid 2–like 2, NF-kB, etc.) (65, 66). Moreover, clinical studies suggest that anthocyanins may improve blood lipid profile by increasing reverse cholesterol transport, regulating HDL functionality, increasing HDL antioxidant capacity, and HDL cholesterol efflux capacity, whereas reducing HDL lipid hydroperoxides (67). Finally, an emerging and growing body of literature is further investigating the role of (poly)phenols and their metabolites on gut microbiota and its potential mediating role on inflammation and prevention of non-communicable diseases (68, 69).

Concerning the comparison between whole fruits and 100% fruit juice, the lack of fiber in the latter is generally considered a limitation from a nutritional point of view. However, the health benefits of fruit appear to go beyond its fiber content, and may instead depend on its overall mineral, vitamin, and possibly (poly)phenol content (70). Only recently, increased attention has been given to the (poly)phenol content of 100% fruit juices as a potential mediator of their health effects (71). A direct comparison of the bioavailability of phenolic compounds in whole fruit and 100% fruit juice suggests that the liquid matrix and lower pectin content of 100% fruit juices could allow for higher intestinal (poly)phenol absorption compared with the solid matrix and higher pectin content of whole fruit (72, 73). Indeed, (poly)phenols are released after a series of mechanical and chemical processes to break down food structure. The ingested molecules in the small intestine are only a small fraction, while the vast majority reach the colon and follow a substantial transformation by the gut microbiota into small-molecular-weight phenolic metabolites, which are ultimately absorbed and further conjugated (74, 75) The whole process seems to be influenced by the food matrix, since the bioavailability of (poly)phenols in whole fruit can be affected by interaction with complex structures (i.e., cell wall or biopolymer interactions), while those in 100% fruit juices might be more easily absorbed even in the small intestine (76). However, it is still unclear what happens to the non-digestible fraction of (poly)phenols reaching the colon and how that affects the gut microbiota and the production of metabolites further absorbed, which could potentially mediate the effects on human health.

There are limitations of the present study that should be considered. First and foremost, data on the (poly)phenol content of 100% fruit juice were available only in a minority of studies. Thus, the overall size effects estimated in the present study may not reflect the entirety of published RCT. However, the aim of this study was not to establish the effects of 100% fruit juice consumption and cardiometabolic risk factors, which have been considered elsewhere (16), but rather to test whether their (poly)phenol content could be considered a mediator for the retrieved effects (in available studies). Second, while the content of a specific (poly)phenol class (i.e., anthocyanins) is more straightforward to compare, the total (poly)phenol content may include a different proportion of the various (poly)phenol classes; given the large variety in chemical composition, pharmacokinetic properties, and mechanisms of action characterizing the different (poly)phenol classes, this approach may not be optimal to determine which bioactive components of 100% fruit juice may be mediating the observed effects on health. Third, related to the above limitation, we could not include other (poly)phenol classes or produce significant analyses due to a lack of data from existing RCT. Fourth, the studies included participants with different health status (i.e., healthy and unhealthy), thus, the effects of the intervention may differ across studies; in fact, we observed stronger size effects when analyzing studies conducted on patients with metabolic syndrome, but residual confounding should be still considered. Fifth, overall diets are generally controlled in both intervention and control groups, but, given the wide variety of foods containing (poly)phenols, there cannot be an absolute exclusion of confounding effects from the external intake of phenolic compounds.

## 5. Conclusion

In conclusion, the present study found that anthocyanins may mediate some of the potential beneficial effects of 100% fruit juices on specific blood lipids. Considering the relevance of this for CVD prevention, it is strongly encouraged that future RCT on 100% fruit juices measure and report the total and specific (poly)phenol content to provide further data to be considered in additional meta-analyses. If these findings are confirmed in future studies, there could be a human health advantage to increasing the (poly)phenol content of 100% fruit juices through the use of specific fruit varieties or targeted plant breeding.

## Data availability statement

The original contributions presented in the study are included in the article/Supplementary material; further inquiries can be directed to the corresponding author.

## Author contributions

AM, PM, and JG contributed to conception and design of the study. AM and JG organized the database. AM performed the statistical analysis. AM, WC, and JG wrote the first draft of the manuscript. AM, WC, CM, AR, IB, AA, DR, PM, and JG wrote the sections of the manuscript. All authors contributed to the article and approved the submitted version.

## Funding

This research was funded by the European Fruit Juice Association (AIJN). AIJN was not involved in the design, conduction, analysis and interpretation of the results.

## Conflict of interest

The authors declare that the research was conducted in the absence of any commercial or financial relationships that could be construed as a potential conflict of interest.

## Publisher’s note

All claims expressed in this article are solely those of the authors and do not necessarily represent those of their affiliated organizations, or those of the publisher, the editors and the reviewers. Any product that may be evaluated in this article, or claim that may be made by its manufacturer, is not guaranteed or endorsed by the publisher.

## Supplementary material

The Supplementary material for this article can be found online at: https://www.frontiersin.org/articles/10.3389/fnut.2023.1175022/full#supplementary-material
