# Supplementary material for: Are (poly)phenols contained in 100% fruit juices mediating their effects on cardiometabolic risk factors? A meta-regression analysis
Source: Front Nutr. 2023 Jun 16;10:1175022. doi: 10.3389/fnut.2023.1175022 (PMC10311565; doi:10.3389/fnut.2023.1175022)
Supplement: Supplementary file 1 [file Data_Sheet_1.docx]

Supplementary Material

**Are (poly)phenols contained in 100% fruit juices mediating their effects on cardiometabolic risk factors? A meta-regression analysis**

Agnieszka Micek*, Walter Currenti, Cristiana Mignogna, Alice Rosi, Ignazio Barbagallo, Ali A. Alshatwi, Daniele Del Rio, Pedro Mena, Justyna Godos

*** Correspondence:** Agnieszka Micek: agnieszka.micek@uj.edu.pl

Supplementary Table 1. PubMed search strategy.

| PubMed search strategy |  |
| --- | --- |
|  | ((juice) AND (lipoprotein[Title/Abstract] OR lipid[Title/Abstract] OR cholesterol[Title/Abstract] OR triglyceride[Title/Abstract] OR HDL[Title/Abstract] OR LDL[Title/Abstract] OR glucose[Title/Abstract] OR insulin[Title/Abstract] OR glycemia[Title/Abstract] OR HOMA[Title/Abstract] OR blood pressure[Title/Abstract] OR blood parameters[Title/Abstract] OR cardiovascular[Title/Abstract] OR hypertension[Title/Abstract] OR diabetes[Title/Abstract] OR dyslipidemia[Title/Abstract] OR cardiometabolic[Title/Abstract] OR metabolic[Title/Abstract] OR cardiometabolic risk factors[MeSH Terms] OR metabolic syndrome[MeSH Terms] OR glucose[MeSH Terms] OR cholesterol[MeSH Terms] OR lipoproteins[MeSH Terms] OR blood pressure[MeSH Terms])) |

Supplementary Table 2. Subgroup analysis on the effect of 100% fruit juice vs. control in randomized controlled trials on cardiovascular risk factors and potential mediating effect of total (poly)phenol content by health status of participants, trial length, and study design. DBP, diastolic blood pressure; HDL-C, high-density lipoprotein-cholesterol; LDL-C, low-density lipoprotein-cholesterol; MD, mean difference; SBP, systolic blood pressure.

|  |  | **Overall effect of juice vs control drink** | | | |  | **Total polyphenol content effect [per 1 g/day]** | | | |
| --- | --- | --- | --- | --- | --- | --- | --- | --- | --- | --- |
| **Subgroup** | **n comparisons** | **MD (95% CI)** | **p** | **I2 [%]** | **pheter** |  | **ΔMD (95% CI)** | **p** | **I2 [%]** | **pheter** |
| ***Total cholesterol*** |  |  |  |  |  |  |  |  |  |  |
| **Health status** |  |  |  |  |  |  |  |  |  |  |
| Low CVD risk | 15 | -0.31 (-4.64; 4.03) | 0.890 | 0.0 | 0.996 |  | -1.42 (-7.57; 4.72) | 0.650 | 0.0 | 0.994 |
| High CVD risk | 12 | -3.26 (-9.59; 3.06) | 0.312 | 18.0 | 0.267 |  | -4.90 (-15.75; 5.95) | 0.376 | 18.1 | 0.271 |
| **Follow-up length** |  |  |  |  |  |  |  |  |  |  |
| <6 weeks | 11 | -2.96 (-8.26; 2.33) | 0.273 | 0.0 | 0.822 |  | -5.05 (-17.12; 7.02) | 0.412 | 0.0 | 0.812 |
| ≥6 weeks | 16 | -1.07 (-5.50; 3.36) | 0.636 | 0.0 | 0.650 |  | -2.15 (-7.94; 3.64) | 0.467 | 0.0 | 0.619 |
| **Study design** |  |  |  |  |  |  |  |  |  |  |
| Crossover | 8 | -2.95 (-7.91; 2.01) | 0.243 | 0.0 | 0.569 |  | -3.80 (-13.76; 6.17) | 0.456 | 0.0 | 0.519 |
| Parallel | 19 | -0.87 (-5.54; 3.79) | 0.713 | 0.0 | 0.822 |  | -2.06 (-8.27; 4.15) | 0.515 | 0.0 | 0.797 |
| ***HDL-c*** |  |  |  |  |  |  |  |  |  |  |
| **Health status** |  |  |  |  |  |  |  |  |  |  |
| Low CVD risk | 12 | 0.30 (-1.54; 2.14) | 0.751 | 0.0 | 0.964 |  | 0.17 (-2.47; 2.82) | 0.899 | 0.0 | 0.940 |
| High CVD risk | 15 | 0.14 (-1.28; 1.57) | 0.844 | 0.0 | 0.949 |  | 0.79 (-1.84; 3.42) | 0.557 | 0.0 | 0.937 |
| **Follow-up length** |  |  |  |  |  |  |  |  |  |  |
| <6 weeks | 12 | 0.26 (-1.81; 2.32) | 0.807 | 0.0 | 0.737 |  | 3.30 (-1.95; 8.55) | 0.218 | 0.0 | 0.797 |
| ≥6 weeks | 15 | 0.18 (-1.17; 1.52) | 0.796 | 0.0 | 0.999 |  | 0.06 (-1.93; 2.05) | 0.955 | 0.0 | 0.998 |
| **Study design** |  |  |  |  |  |  |  |  |  |  |
| Crossover | 7 | 0.46 (-1.54; 2.45) | 0.653 | 0.0 | 0.670 |  | 1.27 (-3.23; 5.76) | 0.581 | 0.0 | 0.587 |
| Parallel | 20 | 0.08 (-1.29; 1.45) | 0.907 | 0.0 | 0.996 |  | 0.36 (-1.69; 2.42) | 0.728 | 0.0 | 0.994 |
| ***LDL-c*** |  |  |  |  |  |  |  |  |  |  |
| **Health status** |  |  |  |  |  |  |  |  |  |  |
| Low CVD risk | 12 | -0.54 (-4.91; 3.84) | 0.810 | 0.0 | 0.957 |  | -3.46 (-10.32; 3.41) | 0.324 | 0.0 | 0.970 |
| High CVD risk | 12 | -3.93 (-8.30; 0.43) | 0.077 | 0.0 | 0.654 |  | -2.42 (-10.56; 5.73) | 0.561 | 0.0 | 0.599 |
| **Follow-up length** |  |  |  |  |  |  |  |  |  |  |
| <6 weeks | 11 | -3.26 (-7.62; 1.10) | 0.143 | 0.0 | 0.873 |  | -3.85 (-14.27; 6.57) | 0.469 | 0.0 | 0.856 |
| ≥6 weeks | 13 | -1.21 (-5.59; 3.17) | 0.588 | 0.0 | 0.744 |  | -3.23 (-9.10; 2.64) | 0.281 | 0.0 | 0.770 |
| **Study design** |  |  |  |  |  |  |  |  |  |  |
| Crossover | 7 | -3.14 (-7.42; 1.14) | 0.151 | 0.0 | 0.541 |  | -12.03 (-24.61; 0.55) | 0.061 | 0.0 | 0.912 |
| Parallel | 17 | -1.26 (-5.73; 3.21) | 0.580 | 0.0 | 0.920 |  | -1.89 (-7.42; 3.64) | 0.503 | 0.0 | 0.908 |
| ***Triglycerides*** |  |  |  |  |  |  |  |  |  |  |
| **Health status** |  |  |  |  |  |  |  |  |  |  |
| Low CVD risk | 13 | 0.33 (-6.17; 6.82) | 0.922 | 0.0 | 0.816 |  | -3.54 (-11.68; 4.61) | 0.395 | 0.0 | 0.809 |
| High CVD risk | 13 | -9.52 (-16.28; -2.76) | 0.006 | 0.0 | 0.995 |  | 1.98 (-15.11; 19.06) | 0.821 | 0.0 | 0.990 |
| **Follow-up length** |  |  |  |  |  |  |  |  |  |  |
| <6 weeks | 11 | 1.79 (-7.09; 10.66) | 0.693 | 0.0 | 0.690 |  | -12.69 (-40.57; 15.19) | 0.372 | 0.0 | 0.681 |
| ≥6 weeks | 15 | -6.80 (-12.31; -1.28) | 0.016 | 0.0 | 0.987 |  | 0.87 (-6.33; 8.08) | 0.812 | 0.0 | 0.978 |
| **Study design** |  |  |  |  |  |  |  |  |  |  |
| Crossover | 8 | -2.11 (-9.85; 5.63) | 0.594 | 0.0 | 0.979 |  | -0.97 (-12.93; 10.98) | 0.873 | 0.0 | 0.955 |
| Parallel | 18 | -5.73 (-11.62; 0.16) | 0.056 | 0.0 | 0.750 |  | -0.73 (-10.00; 8.54) | 0.877 | 0.0 | 0.690 |
| ***Glucose*** |  |  |  |  |  |  |  |  |  |  |
| **Health status** |  |  |  |  |  |  |  |  |  |  |
| Low CVD risk | 11 | -0.55 (-2.36; 1.25) | 0.548 | 40.1 | 0.081 |  | 0.67 (-2.65; 3.98) | 0.694 | 43.3 | 0.069 |
| High CVD risk | 13 | -1.48 (-3.71; 0.76) | 0.195 | 0.0 | 0.582 |  | -0.30 (-4.42; 3.81) | 0.886 | 0.0 | 0.498 |
| **Follow-up length** |  |  |  |  |  |  |  |  |  |  |
| <6 weeks | 11 | -0.84 (-2.82; 1.14) | 0.404 | 11.1 | 0.338 |  | -2.64 (-7.96; 2.69) | 0.332 | 9.7 | 0.353 |
| ≥6 weeks | 13 | -0.79 (-2.69; 1.11) | 0.417 | 25.0 | 0.191 |  | 1.20 (-1.58; 3.98) | 0.398 | 24.4 | 0.204 |
| **Study design** |  |  |  |  |  |  |  |  |  |  |
| Crossover | 9 | -1.51 (-3.27; 0.26) | 0.095 | 26.7 | 0.206 |  | 0.37 (-2.84; 3.58) | 0.821 | 34.4 | 0.154 |
| Parallel | 15 | 0.00 (-1.98; 1.98) | 0.999 | 5.9 | 0.387 |  | 2.41 (-1.61; 6.44) | 0.240 | 2.6 | 0.421 |
| ***SBP*** |  |  |  |  |  |  |  |  |  |  |
| **Health status** |  |  |  |  |  |  |  |  |  |  |
| Low CVD risk | 16 | -1.54 (-3.55; 0.48) | 0.136 | 37.1 | 0.067 |  | 1.38 (-2.57; 5.33) | 0.494 | 39.9 | 0.056 |
| High CVD risk | 11 | -0.13 (-2.63; 2.36) | 0.916 | 0.0 | 0.785 |  | -1.76 (-5.99; 2.48) | 0.416 | 0.0 | 0.770 |
| **Follow-up length** |  |  |  |  |  |  |  |  |  |  |
| <6 weeks | 11 | -0.99 (-3.07; 1.09) | 0.351 | 2.6 | 0.417 |  | -0.31 (-6.87; 6.25) | 0.927 | 12.2 | 0.331 |
| ≥6 weeks | 16 | -1.15 (-3.26; 0.97) | 0.288 | 27.9 | 0.143 |  | 0.49 (-2.80; 3.78) | 0.771 | 32.1 | 0.112 |
| **Study design** |  |  |  |  |  |  |  |  |  |  |
| Crossover | 8 | -1.44 (-3.11; 0.23) | 0.092 | 0.0 | 0.481 |  | -0.05 (-3.19; 3.10) | 0.976 | 8.0 | 0.368 |
| Parallel | 19 | -0.74 (-3.00; 1.53) | 0.524 | 26.3 | 0.141 |  | 1.00 (-3.18; 5.18) | 0.640 | 29.7 | 0.114 |
| ***DBP*** |  |  |  |  |  |  |  |  |  |  |
| **Health status** |  |  |  |  |  |  |  |  |  |  |
| Low CVD risk | 16 | -0.56 (-2.39; 1.27) | 0.550 | 59.1 | 0.001 |  | 1.91 (-1.51; 5.33) | 0.274 | 57.6 | 0.003 |
| High CVD risk | 11 | 0.52 (-1.17; 2.21) | 0.547 | 0.0 | 0.474 |  | -2.14 (-5.54; 1.27) | 0.219 | 0.0 | 0.522 |
| **Follow-up length** |  |  |  |  |  |  |  |  |  |  |
| <6 weeks | 11 | 0.03 (-1.75; 1.80) | 0.978 | 37.8 | 0.098 |  | -1.23 (-6.82; 4.36) | 0.665 | 42.5 | 0.075 |
| ≥6 weeks | 16 | -0.30 (-2.26; 1.65) | 0.761 | 51.0 | 0.010 |  | 1.08 (-1.90; 4.06) | 0.479 | 49.3 | 0.016 |
| **Study design** |  |  |  |  |  |  |  |  |  |  |
| Crossover | 8 | 0.21 (-1.77; 2.19) | 0.836 | 57.0 | 0.023 |  | 1.14 (-2.54; 4.81) | 0.544 | 60.1 | 0.020 |
| Parallel | 19 | -0.48 (-2.29; 1.33) | 0.604 | 40.7 | 0.034 |  | 0.15 (-3.45; 3.75) | 0.934 | 43.7 | 0.025 |

Supplementary Table 3. Subgroup analysis on the effect of 100% fruit juice vs. control in randomized controlled trials on cardiovascular risk factors and potential mediating effect of anthocyanin content by health status of participants, trial length, and study design. DBP, diastolic blood pressure; HDL-C, high-density lipoprotein-cholesterol; LDL-C, low-density lipoprotein-cholesterol; MD, mean difference; SBP, systolic blood pressure.

|  |  | **Overall effect of juice vs control drink** | | | |  | **Anthocyanidin content effect [per 0.1 g/day]** | | | |
| --- | --- | --- | --- | --- | --- | --- | --- | --- | --- | --- |
| **Subgroup** | **n comparisons** | **MD (95% CI)** | **p** | **I^2^ [%]** | **P_heter_** |  | **ΔMD (95% CI)** | **p** | **I^2^ [%]** | **P_heter_** |
| ***Total cholesterol*** |  |  |  |  |  |  |  |  |  |  |
| **Health status** |  |  |  |  |  |  |  |  |  |  |
| Low CVD risk | 16 | -3.63 (-8.14; 0.88) | 0.115 | 5.0 | 0.396 |  | -1.24 (-2.58; 0.10) | 0.069 | 0.0 | 0.567 |
| High CVD risk | 12 | -4.95 (-11.99; 2.08) | 0.168 | 38.1 | 0.087 |  | -2.46 (-6.04; 1.12) | 0.179 | 30.3 | 0.158 |
| **Follow-up length** |  |  |  |  |  |  |  |  |  |  |
| <6 weeks | 15 | -7.72 (-12.85; -2.59) | 0.003 | 18.0 | 0.253 |  | -3.49 (-5.51; -1.48) | 0.001 | 0.0 | 0.963 |
| ≥6 weeks | 13 | -1.30 (-6.58; 3.99) | 0.631 | 10.4 | 0.341 |  | -0.42 (-2.15; 1.31) | 0.632 | 16.3 | 0.284 |
| **Study design** |  |  |  |  |  |  |  |  |  |  |
| Crossover | 10 | -6.67 (-11.43; -1.92) | 0.006 | 2.6 | 0.415 |  | -0.42 (-2.04; 1.21) | 0.616 | 10.7 | 0.346 |
| Parallel | 18 | -3.66 (-9.45; 2.14) | 0.216 | 31.6 | 0.098 |  | -3.35 (-5.41; -1.29) | 0.001 | 0.0 | 0.545 |
| ***HDL-c*** |  |  |  |  |  |  |  |  |  |  |
| **Health status** |  |  |  |  |  |  |  |  |  |  |
| Low CVD risk | 13 | 1.11 (-2.47; 4.69) | 0.542 | 77.3 | <0.001 |  | -0.36 (-1.48; 0.76) | 0.525 | 77.6 | <0.001 |
| High CVD risk | 15 | 0.87 (-1.48; 3.23) | 0.468 | 72.4 | <0.001 |  | 0.32 (-0.81; 1.45) | 0.582 | 73.6 | <0.001 |
| **Follow-up length** |  |  |  |  |  |  |  |  |  |  |
| <6 weeks | 16 | 0.20 (-2.43; 2.84) | 0.879 | 74.6 | <0.001 |  | -0.28 (-1.36; 0.80) | 0.613 | 75.9 | <0.001 |
| ≥6 weeks | 12 | 1.86 (-1.34; 5.06) | 0.255 | 74.8 | <0.001 |  | -0.05 (-1.24; 1.14) | 0.936 | 76.7 | <0.001 |
| **Study design** |  |  |  |  |  |  |  |  |  |  |
| Crossover | 9 | 1.60 (-1.13; 4.33) | 0.252 | 72.6 | <0.001 |  | 0.09 (-0.92; 1.10) | 0.858 | 70.4 | 0.001 |
| Parallel | 19 | 0.62 (-1.95; 3.20) | 0.636 | 66.8 | <0.001 |  | -0.20 (-1.21; 0.81) | 0.697 | 67.4 | <0.001 |
| ***LDL-c*** |  |  |  |  |  |  |  |  |  |  |
| **Health status** |  |  |  |  |  |  |  |  |  |  |
| Low CVD risk | 12 | -2.91 (-7.68; 1.85) | 0.231 | 13.8 | 0.310 |  | -2.72 (-4.59; -0.85) | 0.004 | 0.0 | 0.915 |
| High CVD risk | 12 | -4.37 (-10.95; 2.22) | 0.194 | 46.8 | 0.037 |  | -0.52 (-3.85; 2.80) | 0.757 | 50.2 | 0.029 |
| **Follow-up length** |  |  |  |  |  |  |  |  |  |  |
| <6 weeks | 15 | -5.81 (-11.07; -0.54) | 0.031 | 41.3 | 0.048 |  | -2.63 (-4.44; -0.83) | 0.004 | 12.6 | 0.316 |
| ≥6 weeks | 9 | 0.25 (-5.05; 5.55) | 0.926 | 0.0 | 0.630 |  | -0.01 (-2.49; 2.48) | 0.996 | 0.0 | 0.522 |
| **Study design** |  |  |  |  |  |  |  |  |  |  |
| Crossover | 9 | -4.53 (-10.86; 1.80) | 0.161 | 53.7 | 0.027 |  | -3.44 (-6.99; 0.11) | 0.058 | 39.6 | 0.115 |
| Parallel | 15 | -3.02 (-8.21; 2.16) | 0.253 | 15.4 | 0.281 |  | -1.69 (-3.39; 0.01) | 0.051 | 0.0 | 0.467 |
| ***Triglycerides*** |  |  |  |  |  |  |  |  |  |  |
| **Health status** |  |  |  |  |  |  |  |  |  |  |
| Low CVD risk | 13 | -2.75 (-9.43; 3.93) | 0.420 | 0.0 | 0.633 |  | -0.94 (-2.63; 0.75) | 0.275 | 0.0 | 0.657 |
| High CVD risk | 13 | -13.26 (-18.20; -8.32) | <0.001 | 0.0 | 0.941 |  | -1.44 (-4.81; 1.93) | 0.403 | 0.0 | 0.942 |
| **Follow-up length** |  |  |  |  |  |  |  |  |  |  |
| <6 weeks | 15 | -5.32 (-13.31; 2.66) | 0.191 | 25.4 | 0.174 |  | -3.68 (-6.82; -0.54) | 0.022 | 1.5 | 0.432 |
| ≥6 weeks | 11 | -8.52 (-14.46; -2.58) | 0.005 | 0.0 | 0.992 |  | 0.35 (-1.33; 2.03) | 0.682 | 0.0 | 0.986 |
| **Study design** |  |  |  |  |  |  |  |  |  |  |
| Crossover | 10 | -11.56 (-16.89; -6.24) | <0.001 | 0.0 | 0.546 |  | 0.00 (-1.88; 1.88) | 0.998 | 0.0 | 0.445 |
| Parallel | 16 | -7.02 (-12.98; -1.06) | 0.021 | 0.0 | 0.656 |  | -1.18 (-3.96; 1.61) | 0.407 | 0.0 | 0.637 |
| ***Glucose*** |  |  |  |  |  |  |  |  |  |  |
| **Health status** |  |  |  |  |  |  |  |  |  |  |
| Low CVD risk | 11 | 1.38 (-1.10; 3.85) | 0.276 | 59.9 | 0.005 |  | 0.41 (-0.29; 1.12) | 0.246 | 59.9 | 0.008 |
| High CVD risk | 13 | -1.81 (-4.38; 0.76) | 0.167 | 13.2 | 0.312 |  | -0.43 (-1.70; 0.84) | 0.508 | 14.0 | 0.307 |
| **Follow-up length** |  |  |  |  |  |  |  |  |  |  |
| <6 weeks | 14 | 1.12 (-1.91; 4.14) | 0.470 | 57.5 | 0.004 |  | 0.58 (-0.73; 1.89) | 0.387 | 60.7 | 0.002 |
| ≥6 weeks | 10 | -1.28 (-2.92; 0.35) | 0.124 | 0.2 | 0.435 |  | 0.30 (-0.09; 0.70) | 0.132 | 0.0 | 0.563 |
| **Study design** |  |  |  |  |  |  |  |  |  |  |
| Crossover | 10 | -0.68 (-3.32; 1.96) | 0.613 | 53.8 | 0.021 |  | -0.19 (-1.02; 0.64) | 0.649 | 58.9 | 0.013 |
| Parallel | 14 | 1.22 (-1.52; 3.96) | 0.383 | 39.7 | 0.063 |  | 1.43 (0.62; 2.24) | 0.001 | 0.0 | 0.665 |
| ***SBP*** |  |  |  |  |  |  |  |  |  |  |
| **Health status** |  |  |  |  |  |  |  |  |  |  |
| Low CVD risk | 18 | -0.25 (-2.08; 1.59) | 0.791 | 20.8 | 0.207 |  | 0.12 (-0.49; 0.74) | 0.699 | 24.8 | 0.168 |
| High CVD risk | 11 | -1.59 (-4.16; 0.97) | 0.224 | 8.2 | 0.366 |  | -0.75 (-1.91; 0.42) | 0.208 | 3.1 | 0.411 |
| **Follow-up length** |  |  |  |  |  |  |  |  |  |  |
| <6 weeks | 14 | -0.04 (-1.94; 1.86) | 0.967 | 0.0 | 0.458 |  | 0.80 (-0.27; 1.86) | 0.143 | 0.0 | 0.553 |
| ≥6 weeks | 15 | -1.25 (-3.52; 1.03) | 0.283 | 25.2 | 0.176 |  | -0.26 (-0.97; 0.45) | 0.470 | 29.0 | 0.146 |
| **Study design** |  |  |  |  |  |  |  |  |  |  |
| Crossover | 10 | -1.35 (-3.02; 0.33) | 0.115 | 0.0 | 0.471 |  | -0.13 (-0.70; 0.44) | 0.651 | 5.3 | 0.391 |
| Parallel | 19 | 0.03 (-2.29; 2.35) | 0.980 | 23.4 | 0.172 |  | 0.14 (-0.86; 1.14) | 0.784 | 27.5 | 0.135 |
| ***DBP*** |  |  |  |  |  |  |  |  |  |  |
| **Health status** |  |  |  |  |  |  |  |  |  |  |
| Low CVD risk | 18 | 0.24 (-1.46; 1.95) | 0.779 | 51.3 | 0.006 |  | 0.24 (-0.30; 0.78) | 0.390 | 51.8 | 0.007 |
| High CVD risk | 11 | -0.04 (-1.99; 1.91) | 0.966 | 22.1 | 0.233 |  | -0.63 (-1.44; 0.18) | 0.127 | 11.2 | 0.339 |
| **Follow-up length** |  |  |  |  |  |  |  |  |  |  |
| <6 weeks | 14 | 0.84 (-0.40; 2.09) | 0.184 | 0.0 | 0.493 |  | 0.32 (-0.34; 0.98) | 0.347 | 0.0 | 0.483 |
| ≥6 weeks | 15 | -0.47 (-2.69; 1.74) | 0.676 | 54.5 | 0.006 |  | -0.05 (-0.76; 0.66) | 0.882 | 57.0 | 0.004 |
| **Study design** |  |  |  |  |  |  |  |  |  |  |
| Crossover | 10 | 0.17 (-1.28; 1.63) | 0.815 | 22.3 | 0.238 |  | 0.05 (-0.42; 0.52) | 0.830 | 30.2 | 0.177 |
| Parallel | 19 | 0.21 (-1.81; 2.24) | 0.837 | 50.0 | 0.007 |  | 0.08 (-0.74; 0.90) | 0.847 | 52.5 | 0.005 |

Supplementary Table 4. Overall and subgroup analysis on the effect of 100% fruit juice vs. control in randomized controlled trials on cardiovascular risk factors and potential mediating effect of anthocyanin content by health status of participants, trial length, and study design after exclusion of one study (Loo et al, 2016). DBP, diastolic blood pressure; HDL-C, high-density lipoprotein-cholesterol; LDL-C, low-density lipoprotein-cholesterol; MD, mean difference; SBP, systolic blood pressure.

|  |  | **Overall effect of juice vs control drink** | | | |  | **Anthocyanidin content effect as moderator** | | | |
| --- | --- | --- | --- | --- | --- | --- | --- | --- | --- | --- |
| **Subgroup** | **n comparisons** | **MD (95% CI)** | **p** | **I2 [%]** | **pheter** |  | **MD (95% CI)** | **p** | **I2 [%]** | **pheter** |
| ***Total cholesterol*** |  |  |  |  |  |  |  |  |  |  |
| **Overall** | 27 | -4.74 (-8.81; -0.67) | 0.022 | 24.8 | 0.121 |  | -3.39 (-5.08; -1.70) | <0.001 | 0.0 | 0.793 |
| High CVD risk | 12 | -4.95 (-11.99; 2.08) | 0.168 | 38.1 | 0.087 |  | -2.46 (-6.04; 1.12) | 0.179 | 30.3 | 0.158 |
| Follow-up ≥6 weeks | 12 | -1.26 (-7.19; 4.66) | 0.676 | 17.9 | 0.269 |  | -2.01 (-5.75; 1.74) | 0.294 | 16.6 | 0.286 |
| Crossover design | 9 | -7.15 (-12.35; -1.95) | 0.007 | 6.7 | 0.379 |  | -3.55 (-6.50; -0.61) | 0.018 | 0.0 | 0.887 |
| ***HDL-c*** |  |  |  |  |  |  |  |  |  |  |
| **Overall** | 27 | 1.04 (-0.93; 3.00) | 0.301 | 77.5 | <0.001 |  | -0.09 (-0.92; 0.74) | 0.834 | 77.6 | <0.001 |
| High CVD risk | 15 | 0.87 (-1.48; 3.23) | 0.468 | 72.4 | <0.001 |  | 0.32 (-0.81; 1.45) | 0.582 | 73.6 | <0.001 |
| Follow-up ≥6 weeks | 11 | 2.08 (-1.31; 5.48) | 0.229 | 76.9 | <0.001 |  | 0.32 (-1.51; 2.15) | 0.734 | 79.0 | <0.001 |
| Crossover design | 8 | 1.81 (-1.02; 4.64) | 0.210 | 74.2 | <0.001 |  | 1.59 (0.34; 2.84) | 0.013 | 26.9 | 0.224 |
| ***Triglycerides*** |  |  |  |  |  |  |  |  |  |  |
| **Overall** | 25 | -9.83 (-13.93; -5.73) | <0.001 | 0.0 | 0.631 |  | -2.24 (-4.56; 0.07) | 0.058 | 0.0 | 0.782 |
| High CVD risk | 13 | -13.26 (-18.20; -8.32) | <0.001 | 0.0 | 0.941 |  | -1.44 (-4.81; 1.93) | 0.403 | 0.0 | 0.942 |
| Follow-up ≥6 weeks | 10 | -9.04 (-15.44; -2.64) | 0.006 | 0.0 | 0.987 |  | 0.05 (-4.90; 5.01) | 0.984 | 0.0 | 0.972 |
| Crossover design | 9 | -12.35 (-18.01; -6.70) | <0.001 | 0.0 | 0.514 |  | -4.28 (-8.88; 0.32) | 0.068 | 0.0 | 0.793 |
| ***Glucose*** |  |  |  |  |  |  |  |  |  |  |
| **Overall** | 23 | 0.25 (-1.76; 2.26) | 0.808 | 47.1 | 0.007 |  | 0.64 (-0.26; 1.54) | 0.161 | 47.1 | 0.008 |
| High CVD risk | 13 | -1.81 (-4.38; 0.76) | 0.167 | 13.2 | 0.312 |  | -0.43 (-1.70; 0.84) | 0.508 | 14.0 | 0.307 |
| Follow-up ≥6 weeks | 9 | -1.76 (-3.59; 0.07) | 0.060 | 0.0 | 0.454 |  | 0.96 (-0.32; 2.25) | 0.142 | 0.0 | 0.582 |
| Crossover design | 9 | -0.90 (-4.03; 2.23) | 0.572 | 57.3 | 0.016 |  | -1.89 (-3.60; -0.18) | 0.030 | 40.0 | 0.112 |
| ***SBP*** |  |  |  |  |  |  |  |  |  |  |
| **Overall** | 28 | -0.57 (-2.12; 0.97) | 0.469 | 17.2 | 0.210 |  | 0.16 (-0.65; 0.97) | 0.697 | 19.7 | 0.181 |
| High CVD risk | 11 | -1.59 (-4.16; 0.97) | 0.224 | 8.2 | 0.366 |  | -0.75 (-1.91; 0.42) | 0.208 | 3.1 | 0.411 |
| Follow-up ≥6 weeks | 14 | -1.03 (-3.59; 1.53) | 0.430 | 30.2 | 0.135 |  | -0.54 (-1.88; 0.79) | 0.423 | 33.6 | 0.114 |
| Crossover design | 9 | -1.19 (-3.07; 0.69) | 0.214 | 5.9 | 0.386 |  | -1.52 (-6.10; 3.06) | 0.515 | 14.5 | 0.317 |
| ***DBP*** |  |  |  |  |  |  |  |  |  |  |
| **Overall** | 28 | 0.08 (-1.26; 1.43) | 0.903 | 43.2 | 0.009 |  | 0.04 (-0.59; 0.68) | 0.898 | 45.2 | 0.006 |
| High CVD risk | 11 | -0.04 (-1.99; 1.91) | 0.966 | 22.1 | 0.233 |  | -0.63 (-1.44; 0.18) | 0.127 | 11.2 | 0.339 |
| Follow-up ≥6 weeks | 14 | -0.56 (-3.01; 1.88) | 0.652 | 55.6 | 0.006 |  | -0.44 (-1.68; 0.80) | 0.490 | 58.6 | 0.004 |
| Crossover design | 9 | 0.09 (-1.58; 1.76) | 0.915 | 29.9 | 0.180 |  | -0.78 (-3.76; 2.20) | 0.608 | 37.9 | 0.127 |

Supplementary Figure 1. Risk of bias of the randomized controlled trials included in the analyses.


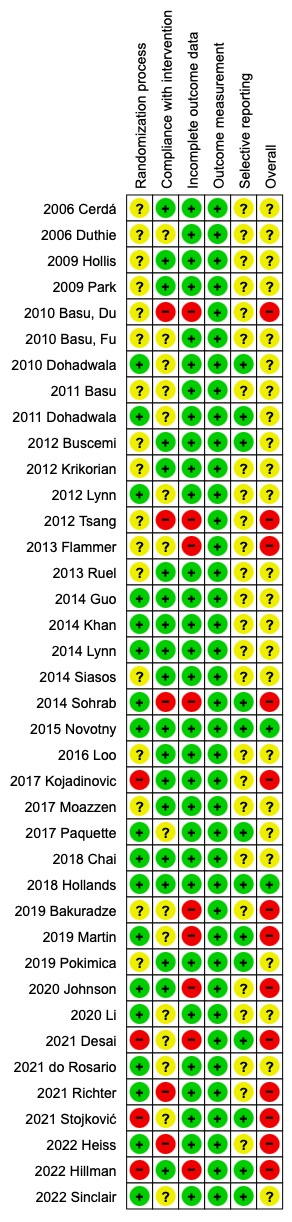


Supplementary Figure 2. Summary risk of bias of the randomized controlled trials included in the analyses.


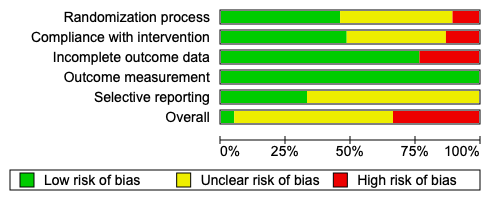


Supplementary Figure 3. Effect of 100% fruit juice vs. control in randomized controlled trials reporting content of (poly)phenol in intervention group on cardiovascular risk factors: A) Total cholesterol [mg/dL], B) HDL-c [mg/dL], high-density lipoprotein-cholesterol, C) LDL-c [mg/dL], low-density lipoprotein-cholesterol; D) Triglycerides [mg/dL], E) Glucose [mg/dL], F) DBP [mmHg], diastolic blood pressure; G) SBP [mmHg], systolic blood pressure. MD, mean difference.


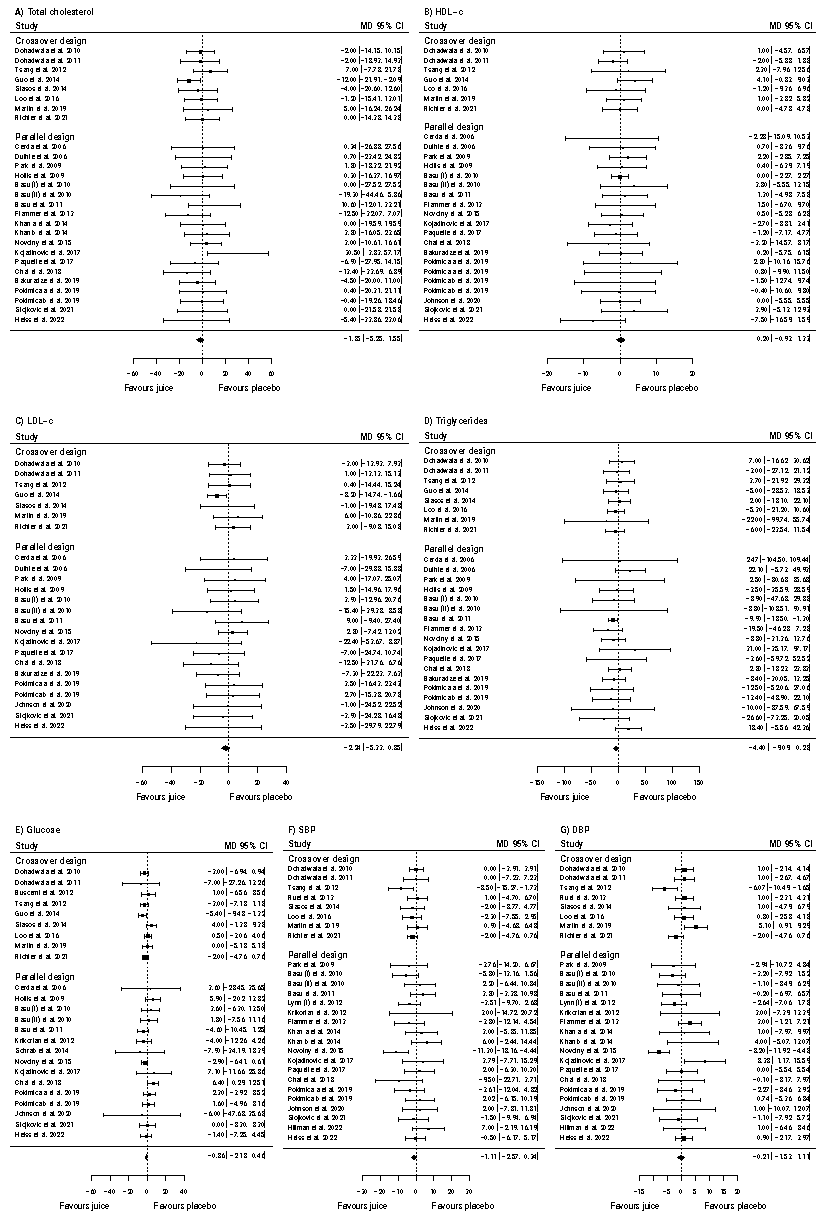


Supplementary Figure 4. The funnel plots for the effect of 100% fruit juice vs. control in randomized controlled trials reporting content of (poly)phenol in intervention group on cardiovascular risk factors. Figures visualize small-study effects (showing the relationship between the precision and observed effect size of studies) as the possible indicators of publication bias. Presented p values refer to the quantitative assessment of the presence of funnel plot asymmetry through Egger’s regression test.

Supplementary Figure 5. Effect of 100% fruit juice vs. control in randomized controlled trials reporting content of anthocyanins in intervention group on cardiovascular risk factors: A) Total cholesterol [mg/dL], B) HDL-c [mg/dL], high-density lipoprotein-cholesterol, C) LDL-c [mg/dL], low-density lipoprotein-cholesterol; D) Triglycerides [mg/dL], E) Glucose [mg/dL], F) DBP [mmHg], diastolic blood pressure; G) SBP [mmHg], systolic blood pressure. MD, mean difference.


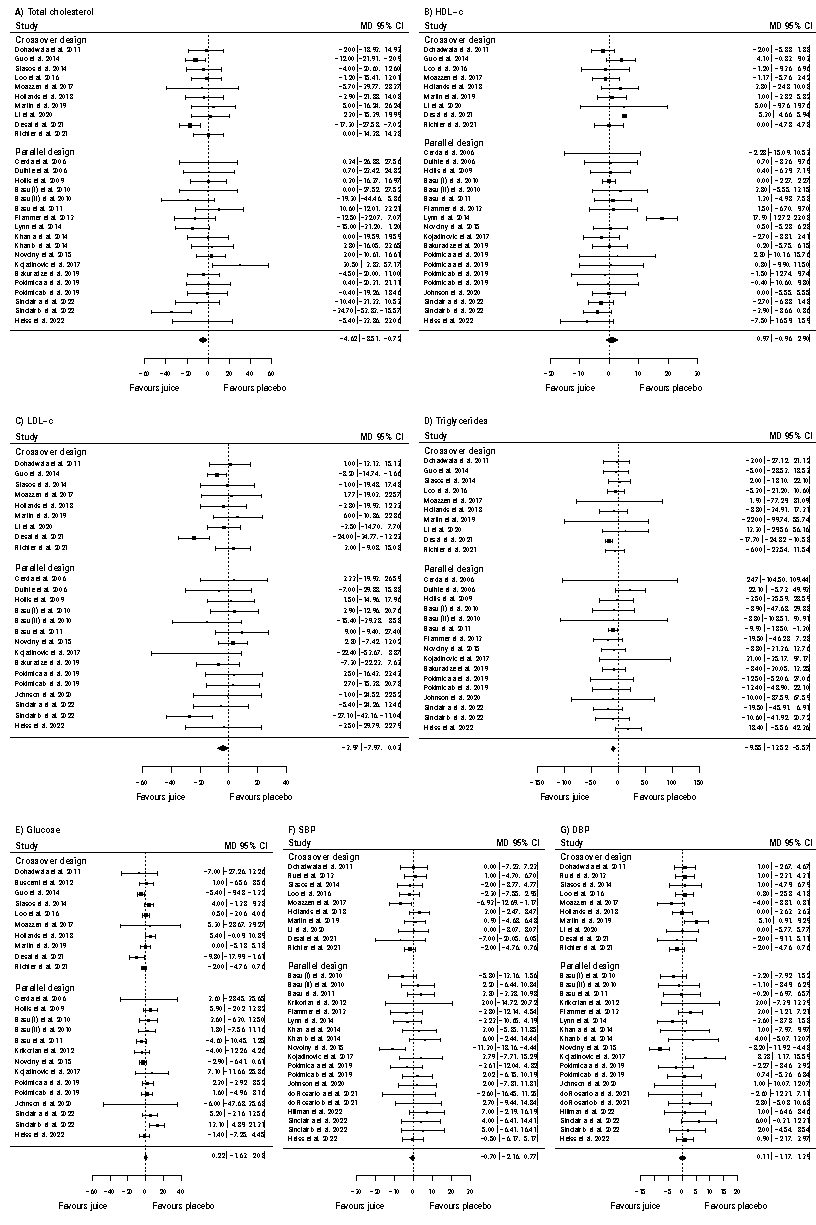


Supplementary Figure 6. The funnel plots for the effect of 100% fruit juice vs. control in randomized controlled trials reporting content of anthocyanins in intervention group on cardiovascular risk factors. Figures visualize small-study effects (showing the relationship between the precision and observed effect size of studies) as the possible indicators of publication bias. Presented p values refer to quantitative assessment of the presence of funnel plot asymmetry through Egger’s regression test.
